# The α_1D_-adrenergic receptor is expressed intracellularly and coupled to increases in intracellular calcium and reactive oxygen species in human aortic smooth muscle cells

**DOI:** 10.1186/1750-2187-3-6

**Published:** 2008-02-27

**Authors:** Mary L García-Cazarín, Jennifer L Smith, Kyle A Olszewski, Dan F McCune, Linda A Simmerman, Robert W Hadley, Susan D Kraner, Michael T Piascik

**Affiliations:** 1 Department of Molecular and Biomedical Pharmacology, University of Kentucky; Lexington, KY USA; 2 The Nesbitt School of Pharmacy, Department of Pharmaceutical Sciences, Wilkes University; Wilkes, PA USA

## Abstract

**Background:**

The cellular localization of the α_1D_-adrenergic receptor (α_1D_-AR) is controversial. Studies in heterologous cell systems have shown that this receptor is expressed in intracellular compartments. Other studies show that dimerization with other ARs promotes the cell surface expression of the α_1D_-AR. To assess the cellular localization in vascular smooth muscle cells, we developed an adenoviral vector for the efficient expression of a GFP labeled α_1D_-AR. We also measured cellular localization with immunocytochemistry. Intracellular calcium levels, measurement of reactive oxygen species and contraction of the rat aorta were used as measures of functional activity.

**Results:**

The adenovirally expressed α_1D_-AR was expressed in intracellular compartments in human aortic smooth muscle cells. The intracellular localization of the α_1D_-AR was also demonstrated with immunocytochemistry using an α_1D_-AR specific antibody. RT-PCR analysis detected mRNA transcripts corresponding to the α_1A_-α_1B_- and α_1D_-ARs in these aortic smooth muscle cells. Therefore, the presence of the other α_1_-ARs, and the potential for dimerization with these receptors, does not alter the intracellular expression of the α_1D_-AR. Despite the predominant intracellular localization in vascular smooth muscle cells, the α_1D_-AR remained signaling competent and mediated the phenylephrine-induced increases in intracellular calcium. The α_1D_-AR also was coupled to the generation of reactive oxygen species in smooth muscle cells. There is evidence from heterologous systems that the α_1D_-AR heterodimerizes with the β_2_-AR and that desensitization of the β_2_-AR results in α_1D_-AR desensitization. In the rat aorta, desensitization of the β_2_-AR had no effect on contractile responses mediated by the α_1D_-AR.

**Conclusion:**

Our results suggest that the dimerization of the α_1D_-AR with other ARs does not alter the cellular expression or functional response characteristics of the α_1D_-AR.

## Background

The α_1_-ARs are members of the class I of the G-protein coupled receptors (GPCR) superfamily [1,2]. Three α_1_-ARs, α_1A_-AR, α_1B_-AR and α_1D_-AR have been cloned and characterized [1,3,4]. These receptors mediate responses to epinephrine and norepinephrine thus making a vital contribution to the control of blood flow and systemic arterial blood pressure. Abnormalities in the regulation of the α_1_-ARs may contribute to the development of hypertension and heart failure [5-8].

It is well known that the localization and trafficking properties of a receptor can modulate its physiological function [9]. Results from heterologous expression systems have demonstrated that the α_1B_-AR is localized on the cell surface, as expected for a GPCR, while the α_1A_-AR is localized not only on the cell but also in intracellular compartments [10,11]. In contrast, we have shown that the α. In contrast, we have shown that the α_1D_-AR is localized intracellularly [11,12]. These results of nonconical cellular locatization are consistent with emerging data that show specific GPCR families can be localized not only to intracellular sites but also on the nuclear membrane [13].

In recent years, the concept of receptor dimerization has brought a new perspective to GPCR function [14-16]. Previous studies reported that the α_1D_-AR interacts with the α-AR interacts with the α_1B_-AR and the β_2_-AR [17] resulting in the cell surface expression of the α_1D_-AR. This has lead to the suggestion that these receptors are capable of heterodimerization [17-20]. These observations have been made in heterologous systems. However, the role of dimerization in the regulation of cells that natively express all three receptors such as vascular smooth muscle cells has not been well studied. This is due in part to the difficulty of tranfecting smooth muscle cells. To overcome this obstacle, we developed a recombinant adenovirus for the efficient expression of the human α_1D_-AR. We show that despite the presence of the other α_1_-AR family members, the α_1D_-AR is expressed mainly in intracellular compartments. We further show that while receptor dimerization may occur, it does not appear to alter the functional properties of the α_1D_-AR.

## Results

### Cellular localization

**Figure 1:**
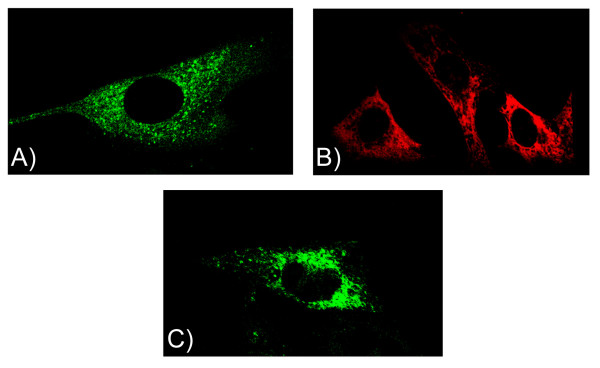
**The Cellular Localization of the α_1D_-AR in Human Aortic Smooth Muscle Cells**. Panel A; Human aortic smooth muscle cells were infected with an α_1D_-AR/GFP expressing virus. Panel B; Immunofluorescence localization of the α_1D_-AR in human aortic smooth muscle cells. Panel C; Rat 1 Fibroblasts were infected with an α_1D_-AR/GFP expressing virus. Adenoviral infection and immunostaining were carried out as described in Methods. Localization patterns were visualized with laser scanning confocal microscopy.

An adenoviral vector was constructed to drive the efficient expression of a GFP-labeled α_1D_-AR. Infection of aortic smooth muscle cells with virus expressing the α_1D_-AR/GFP resulted in approximately 80% receptor transfectional efficiency (not shown) demonstrating that adenovirus can be useful in cells that have been traditionally difficult to transfect with the α_1_-ARs. Following viral infection, the α_1D_-AR/GFP was detected in intracellular compartments of aortic smooth muscle cells (Figure 1A). A similar pattern of vascular smooth muscle intracellular expression was seen with immunocytochemistry studies using an α_1D_-AR selective antibody (Figure 1B). These localization results in smooth muscle cells are similar to our previous findings in HEK 293 cells transfected with an α_1D_-AR/GFP expression plasmid or immunohistochemistry studies in fibroblasts stably transfected with the α_1D_-AR [12,21]. Recently, it was proposed that the α_1D_-AR can dimerize with the α-AR can dimerize with the α_1B_-AR promoting its cell surface expression [19]. Our results would argue that the presence of other ARs, particularly the α_1B_-AR, does not alter α_1D_-AR localization in vascular smooth muscle cells. To further substantiate that the presence of the α_1B_-AR does not affect α_1D_-AR localization, we infected fibroblasts that stably express the α_1B_-AR with the α-AR with the α-AR with the α-AR with the α_1D_-AR/GFP adenoviral construct (Figure 1C). Despite expression in a cell expressing the α_1B_-AR at high levels, α_1D_-AR was nonetheless expressed in intracellular compartments (Figure 1C). These data argue that the α_1B_-AR does not alter the cellular localization of the α_1D_-AR.

**Figure 2:**
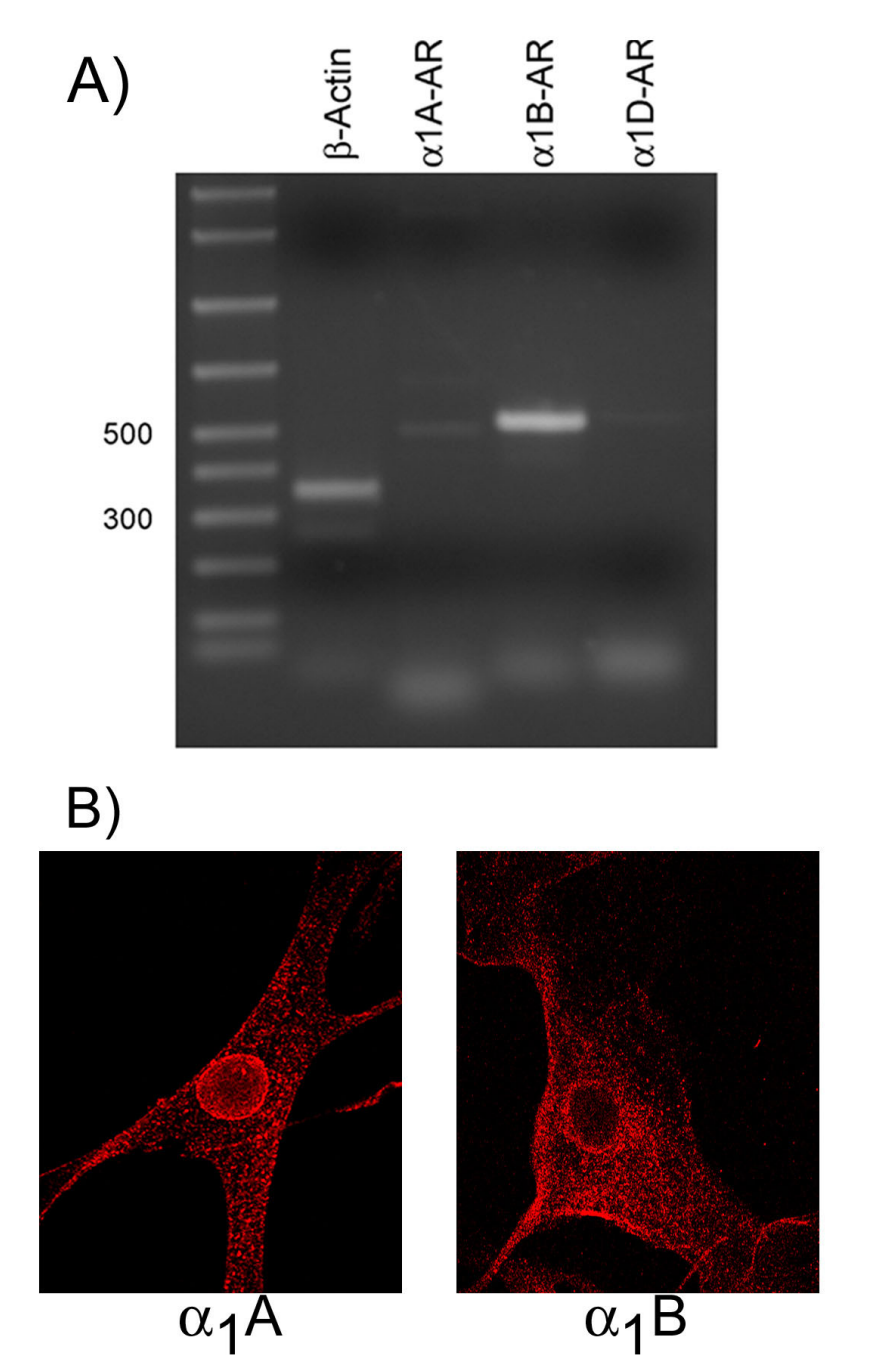
***Panel A*; **RT-PCR Measurement of α_1_-AR mRNA in Human Aortic Smooth Muscle cells. ***Panel B*; **Immunolocalization of the α_1_-ARs in human aortic smooth muscle cells. Reverse transcription and PCR analysis and immunocytochemistry were performed as described in Methods.

It is possible, however, that in these cultured smooth muscle cells the α_1B_-AR and/or α_1A_-AR are not expressed. To examine this possibility we used RT-PCR to assess message expression. The results of these experiments are shown in figure 2A. A very prominent PCR-product corresponding to the α_1B_-AR was detected in aortic smooth muscle cells. We were also able to detect an α_1A_- AR transcript. A very faint product corresponding to the α- AR transcript. A very faint product corresponding to the α_1D_-AR was also observed. Minute levels of tissue expression are typical for this receptor. A series of antibodies directed against each of the α_1_-ARs was used to determine the cellular localization of these receptors (Figure 2B). As has been shown by previous work the α). As has been shown by previous work the α_1A_- AR is expressed both intracellularly as well as on the cell surface while the α_1B_-AR is expressed predominately on the cell membrane. What is also apparent from comparing figures 1A–C and figure 2B is that the expression pattern of the α is that the expression pattern of the α_1D_- AR is markedly different from that of either the α- AR is markedly different from that of either the α_1A_- or α- or α_1B_-ARs. Therefore, in a mammalian cell where both the α_1A_- and α- and α_1B_-AR are natively expressed (as opposed to being transfected) the α_1D_-AR is nonetheless expressed intracellularly. Further, the data argue that while dimerization may occur in smooth muscle cells, it does not alter the localization of the α_1D_-AR.

### Effects on intracellular calcium

**Figure 3:**
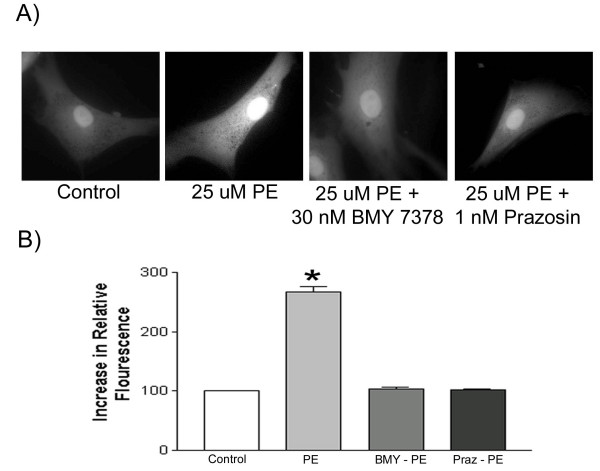
**Effect of Phenylephrine on Intracellular Calcium Levels in Human Aortic Smooth Muscle Cells**. Human aortic smooth muscle cells were loaded with Calcium Green 1 AM for 1 hr. The ability of 25 uM phenylephrine to increase intracellular calcium was studied alone and following treatment with either 1 nM prazosin or 30 nM BMY 7378. Experiments were carried out as described in Methods. The results of a typical imaging study are presented along with a graphical summary of the statistical analysis of four independent experiments. Data were analyzed by a one-way ANOVA followed by post hoc testing. * Indicates a statistically significant difference from the untreated control.

**Figure 4:**
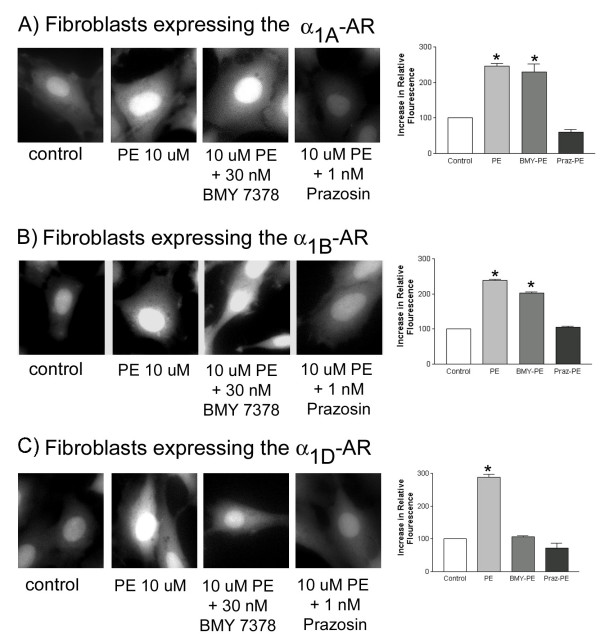
**Effect of Phenylephrine on Intracellular Calcium Levels in Stably Transfected Rat 1 Fibroblasts**. Rat 1 Fibroblasts, stably transfected with each of the α_1_-ARs, were loaded with Calcium Green 1 AM for 1 hr. The ability of 10 uM phenylephrine to increase intracellular calcium was studied alone and following treatment with either 1 nM prazosin or 30 nM BMY 7378. Experiments were carried out as described in Methods. The results of a typical imaging study are presented along with a graphical summary of the statistical analysis of four independent experiments. Data were analyzed by a one-way ANOVA followed by post hoc testing. * Indicates a statistically significant difference from the untreated control.

In aortic smooth muscle cells phenylephrine produced a dose-dependent and statistically significant increase in intracellular calcium (see data for 25 uM presented in Figure 3). This increase was antagonized by 1 nM of the nonselective α_1_-AR blocker prazosin or 30 nM of the highly selective α_1D_-AR antagonist BMY 7378. To substantiate the selectivity of this dose of BMY 7378 we measured phenylephrine-induced increases in intracellular calcium levels in fibroblasts stably transfected with either the α_1A_-AR, α_1B_-AR or the α_1D_-AR. Despite pretreatment with 30 nM BMY 7378, phenylephrine maintained the ability to promote increases in intracellular calcium in the α_1A_-AR or α_1B_-AR expressing lines of fibroblasts (Figure 4). In contrast, BMY 7378 blocked the phenylephrine-induced increases in intracellular calcium in fibroblasts stably transfected with the α_1D_-AR (Figure 4). Therefore, BMY 7378 at 30 nM, the concentration used in the vascular smooth muscle cells (see above), can selectively block the α_1D_-AR. These data support the conclusion that the receptor that mediates increases in intracellular calcium in aortic smooth muscle cells is the α_1D_-AR.

### Effects on reactive oxygen species

**Figure 5:**
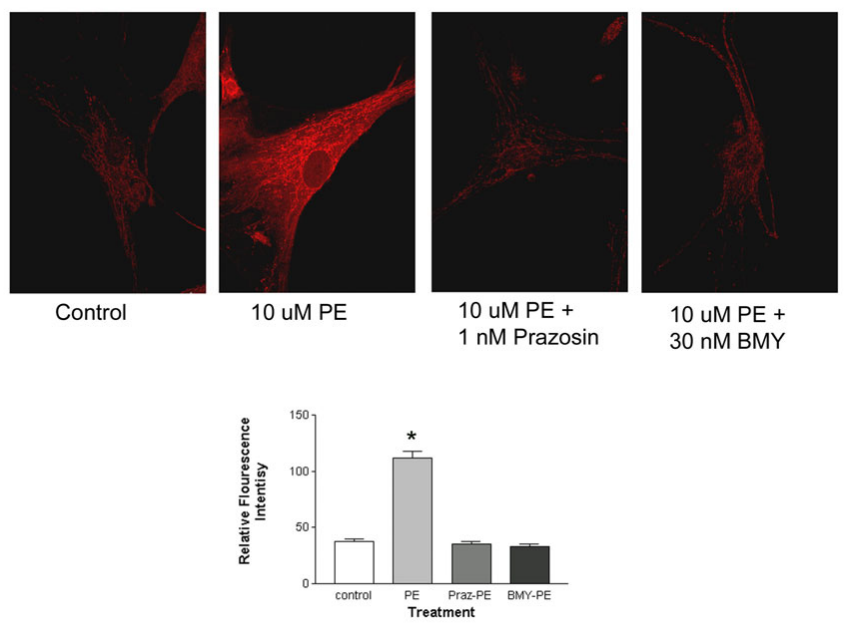
**Effect of Phenylephrine on the Levels of Reactive Oxygen Species Levels in Human Aortic Smooth Muscle Cells**. Human aortic smooth muscle cells were loaded with Mitotracker ROS for 20 min. The ability of 10 uM phenylephrine to increase the levels of reactive oxygen species was studied alone and following treatment with either 1 nM prazosin or 30 nM BMY 7378. Experiments were carried out as described in Methods. The results of a typical imaging study are presented along with a graphical summary of the statistical analysis of four independent experiments. Data were analyzed by a one-way ANOVA followed by post hoc testing. * Indicates a statistically significant difference from the untreated control.

This type of specificity of coupling was also observed using a novel functional response to activation of the α_1_-AR-namely the generation of reaction oxygen species. In human aortic smooth muscle cells, phenylephrine produced a rapid, dose-dependent and statistically significant increase in the level reactive oxygen species (Figure 5). While we present the data with 10 uM, we could see statistically significant increases in ROS at 1 uM phenylephrine. This increase was blocked by 1 nM prazosin or 30 nM BMY 7378. Therefore, it is the α_1D_-AR that mediates increases in reactive oxygen species in these vascular smooth muscle cells.

### Effects on smooth muscle contraction

Recent data from heterologous systems have suggested that the interaction between the α_1D_-AR and other G-protein coupled receptors alters the pharmacologic properties of the α_1D_-AR [17,19]. Our results from vascular smooth muscle cells that naturally express all three receptors indicate that functional responses from a receptor with α_1D_-AR characteristics can be detected.

**Figure 6:**
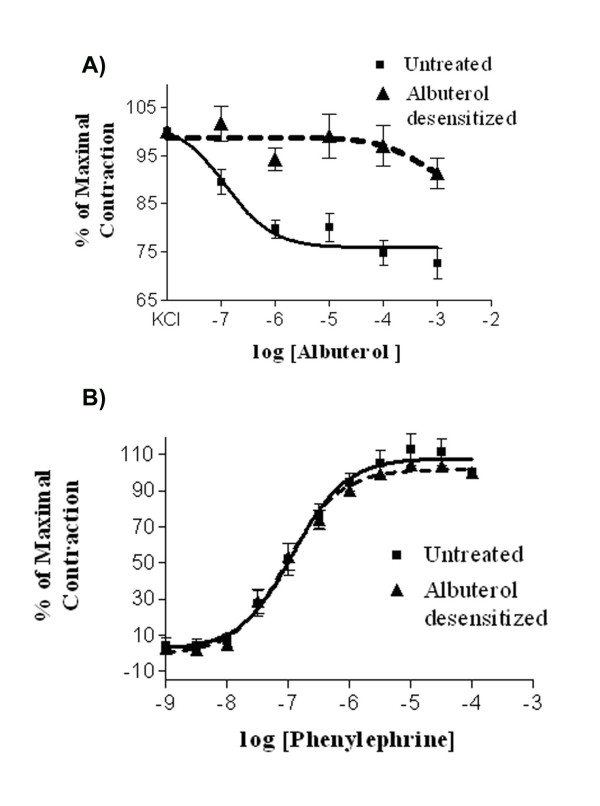
**Effect of the Albuterol Pretreatment of Phenylephrine-Induced Contraction of the Rat Aorta**. Panel A; Log-dose response curves of the ability of albuterol to relax the rat aorta. The aorta was contracted with KCl and the albuterol responses were assessed in control blood vessels and in those vessels desensitized by incubation with 1 uM albuterol for 12 hr. Data are the mean +/- the SEM of 10 (control) or 13 (desensitized) experiments. Panel B; Log-dose response curves for the ability of phenylephrine to induce contraction of the aorta following desensitization with albuterol. Data are the mean +/- the SEM of 6 independent experiments.

To assess the relevance of the interaction between the α_1D_-AR and the other ARs in an intact blood vessel system, we studied contractile responses in the rat aorta. In previous work we have shown that the contractions of the rat aorta are mediated by the α_1D_-AR [22]. In addition to potential dimerization among the α_1_-ARs, there is evidence of heterodimerization between the α_1D_-AR and β_2_-AR. Studies in heterologous systems also show that desensitization of the β_2_-AR with albuterol promotes the internalization and desensitization of the α_1D_-AR [17]. We assessed responses in the rat aorta following a 12 hr exposure to albuterol. After this incubation period, the responses to albuterol were significantly decreased when compared to vehicle treated aorta (Figure 6). This indicates a desensitization of the β_2_-AR mediated response. The phenylephrine log dose response curves were the same in control and albuterol desensitized aorta. Therefore, desensitization of β_2_-AR does not lead to desensitization of the α_1D_-AR.

## Discussion

Previous work from our laboratory has shown that in heterologous systems the α_1D_-AR localizes intracellularly and does not undergo agonist-mediated internalization or desensitization [11,12,21]. Due in part to problems with transfection, it has been difficult to determine if this type of expression pattern occurs in cells that natively express the α_1D_-AR along with the other α_1_-AR family members. To facilitate its efficient expression, we developed an adenoviral vector expressing the human α_1D_-AR fused with the GFP. We then used the vector to infect human aortic smooth muscle cells. RT-PCR analysis showed that these cells express all three α_1_-ARs (Figure 2). The rank order of mRNA expression was α_1B_-AR> α_1A_-AR>>α_1D_-AR. This type of expression pattern is typical for the α_1_-ARs. Following infection of aortic smooth muscle cells we observed that the α_1D_-AR was localized to intracellular compartments (Figure 1A). An intracellular localization pattern was also observed when aortic smooth muscle cells were immunostained with an α_1D_-AR antibody (Figure 1B). Therefore, while dimerization between the α_1D_-AR and the other α_1_-ARs may occur in aortic smooth muscle cells, this does not alter the cellular localization of the α_1D_-AR.

If the α_1D_-AR forms heterodimers with the other α_1_-ARs, then it is possible that these complexes exhibit properties different from the α_1D_-AR alone [17,19]. We examined this possibility using the selective antagonist BMY 7378. In previous work we calculated that at 30 nM over 90 % of the α_1D_-ARs would be occupied by BMY 7378 while less than 10 % of either the α_1A_-AR or the α_1B_-AR would be occupied by this antagonist [22]. Therefore, at this concentration BMY 7378 would be anticipated to be highly selective for the α_1D_-AR. This was substantiated in fibroblasts stably transfected with each of the α_1_-ARs. In these fibroblast cell lines phenylephrine treatment produced a significant increase in intracellular calcium. However, only in fibroblasts expressing the α_1D_-AR was BMY 7378 capable of antagonizing the calcium response to phenylephrine, indicating that BMY 7378 is selective for the α_1D_-AR (Figure 4). The phenylephrine-mediated increases in intracellular calcium in aortic smooth muscle cells were also blocked by this dose of BMY 7378 (Figure 3). In a similar fashion, we demonstrated that the generation of reactive oxygen species in aortic smooth muscle cells was antagonized by 30 nM BMY 7378 (Figure 5). There is no evidence that BMY 7378 at this concentration can block either the α_1A_- or the α_1B_-AR. Therefore the antagonism seen with BMY 7378 indicates that the observed increases in intracellular calcium and reactive oxygen species are mediated by a receptor of α_1D_-AR character. In aggregate, the data suggest that while heterodimerization may occur, it does not appear to alter the pharmacologic properties of the α_1D_-AR. We know that both increases in intracellular calcium and elevations in ROS are mediated by the α_1D_-AR. What we do not know is if the intracellularly expressed α_1D_-AR is signaling competent and responsible for these effects or whether it is a small population of cell surface expressed receptors. If signaling does indeed emanate from the intracellular α_1D_-AR, then there has to be a pathway that would allow agonist access to these receptors.

In addition to dimerization within the α_1_-AR family, there is also evidence that the α_1D_-AR can form dimers with the β_2_-AR. Studies in expression systems have shown that not only does the β_2_-AR promote the cell surface expression of the α_1D_-AR but that desensitization of the β_2_-AR also desensitized the α_1D_-AR response. We wished to determine if this type of activity could be obtained in a functional system that natively expresses these receptors without resorting to overexpression of cloned receptors in a model cell system. The contractile responses of phenylephrine in the rat aorta are due to interactions at the α_1D_-AR (see for example, Piascik et al. [22]). Therefore, we assessed potential β_2_-AR/α_1D_-AR interactions using this blood vessel. Overnight treatment of blood vessels with albuterol caused desensitization of the β_2_-AR as shown by diminished vasodilatory responses to this agent (Figure 6A). However, desensitization of the β_2_-AR did not cause a rightward shift of the phenylephrine dose response curve (see Figure 6B). Therefore, desensitization of the β_2_-AR does not alter contractile responses to the α_1D_-AR. These results show that cross desensitization between β_2_-AR and the α_1D_-AR does not occur in an intact, responding segment of vascular smooth muscle.

## Conclusion

In summary, adenoviral vectors expressing the α_1_-ARs are a novel and efficient tool to investigate properties of these receptors in native cells. These vectors were used to show that in human smooth muscle cells expressing all three α_1_-ARs, the α_1D_-AR is localized in intracellular compartments. Therefore, despite recent reports of heterodimerization between the α_1_-ARs, in a human vascular smooth muscle cell line, the α_1D_-AR is still expressed intracellularly. Indeed none of the data we obtained in this work support the idea of α_1D_-AR heterodimerization. Using three independent measures (calcium levels, generation of reactive oxygen or vascular smooth muscle contraction) we also could not detect any evidence of altered pharmacologic properties of the α_1D_-AR.

## Methods

### Cell culture conditions

Human aortic smooth muscle cells were obtained from Cascade Biologics (Portland, OR) and grown in Medium 231 supplemented with smooth muscle growth supplement until they become confluent (Cascade Biologics, Portland, OR). Stably transfected Rat 1 fibroblast lines expressing each of the α_1_-AR subtypes were maintained in Dulbecco's modified Eagle's medium (Cellgro, Herdon, VA) supplemented with 10% fetal bovine serum and a 1% antibiotic/antimycotic cocktail (Invitrogen, Carlsbad, CA). All cells were grown in T75 flasks in a 37°C cell culture incubator with a humidified atmosphere (95% air and 5% CO_2_) and were fed every 2 to 3 days. After reaching confluence the cells were plated on plain untreated coverslips in 35 mm tissue culture dishes.

### Construction of recombinant adenoviruses expressing the α_1_-ARs

A vector expressing the human α_1D_-AR coupled to the green fluorescent protein (α_1D_-AR/GFP) was provided by Dr. Gozoh Tsujimoto [23,24]. This vector was digested with *EcoR*I and *Xba*I enzymes and cloned into the pCI expression vector (Promega. Madison, WI). pCI was digested with *Bgl*II and *Cla*I. This fragment included the CMV I.E. promoter, the α_1D_-AR/GFP and the SV40 late poly (A). The *Bgl*II and *Cla*I fragment was cloned into the adenovirus recombination vector pAdLink. pAdLink and the wild type adenovirus vector, dl327, were linearized with *Nhe*I and *Cla*I respectively. Homologous recombination occurred by co-transfecting linearized pAdLink and the wild type adenovirus vector into HEK 293 cells. Positive plaques appeared 10 to 14 days after recombination and were then amplified. Plaques were purified by serial dilutions of a positive plaque (usually from 10^-3 ^to 10^-12^) in 96 well plates using HEK 293 cells. After plaque purification, samples of viral DNA were analyzed for wild type virus contamination by PCR [25]. Once a purified adenovirus was obtained, the plaque was amplified for large-scale production. Fifty 150 mm dishes of HEK 293 cells were used for amplification of adenovirus which was then purified using double cesium gradients. Adenovirus was tittered using the Adeno-X™ Rapid Titer Kit from BD Biosciences (Palo Alto, CA).

### Infection of cells with recombinant adenovirus

Human aortic smooth muscle cells *or Rat 1 fibroblasts *were grown on glass coverslips. Two hours prior to infection, cells were placed in serum free medium and infected with adenovirus. Twenty four hours after infection the medium was changed and the virus free incubation was allowed to proceed for an additional 24 hours. Cells expressing the GFP labeled α_1D_-AR were fixed with 3.7% Formaldehyde in PBS for 10 mins. Cells were then mounted on slides with Vectashield (Vector Labs, Burlingame, CA). Cells were visualized using a Leica TCS SP 5 AOBS confocal microscope with a Plan-Apo 64X oil immersion objective lens (Leica, Wetzlar, Germany) using Leica TCS NT version 2.5 software. Images were transferred to a computer for reduction with Adobe Photoshop version 6.0 (Adobe Systems, Mountain View, CA).

### Immunocytochemistry

Human aortic smooth muscle cells or grown on glass cover slips, were washed in PBS and fixed with 3.7% Formaldehyde in PBS for 10 min. Cells were then washed with .05% BSA in PBS and permeabilized with 0.1% Triton in PBS for 5 min. After permeabilization, the cells were washed and blocked with 10% lamb serum for 1 hour at room temperature. After washing polyclonal antibodies (Affinity Bioreagents, Golden, CO) against each of the α_1_-ARs, diluted 1:100 in 1% BSA in PBS, was added and incubated overnight at 4°C. Following this incubation, the cells were washed with .05% BSA in PBS and a Texas Red secondary antibody (Abcam, Cambridge MA), diluted 1:500 in PBS, was added and incubated in the dark at room temperature for 1 hr. Cells were washed with PBS and mounted on glass slides with Vectashield (Vector Laboratories, Burlingame, CA). Cells were visualized with a confocal microscope as described above.

### RT-PCR

Total RNA from HASMCs was isolated and purified with the ChargeSwitch Total RNA kit from Invitrogen (Carlsbad, CA), and 0.5 μg samples were reverse transcribed at 45°C for one hour using Cloned Avian Myeloblastosis Virus (AMV) Reverse Transcriptase and Oligo(dT)_20 _primer (Invitrogen, Carlsbad, CA). After heating at 85°C for 5 min. to terminate the reaction, cDNA samples were stored at -20°C until used. Negative controls for the presence of genomic DNA were performed by replacing the reverse transcriptase enzyme with *Taq *DNA polymerase.

For PCR, primers were synthesized by Invitrogen based on those used by Esbenshade *et al*. [26] to detect and distinguish specific human AR subtypes. Sequences of the primers were as follows: α_1A_, 5'-ATGCTCCAGCCAAGAGTTCA-3' (sense, annealing to bases 1417–1437) and 5'-TCCAAGAAGAGCTGGCCTTC-3' (antisense, bases 1898–1918); α_1B_, 5'-CTGTGCGCCATCTCCATCGATCGCTAC-3' (sense, bases 406–432) and 5'-ATGAAGAAGGGTAGCCAGCACAAGATGAA-3' (antisense, bases 907–935); α_1D_, 5'-CTCTGCACCATCTCCGTGGACCGGTAC-3' (sense, bases 563–589) and 5'-AAAGAAGAAAGGGAACCAGCAGAGCACGAA-3' (antisense, bases 1073–1102). The receptor specific primers target sequences within the third intracellular loop (sense) and the carboxy terminus (antisense). Primers for β-actin were included as a positive control (RT-PCR Primer and Control Set, Invitrogen). The predicted sizes of the amplified human β-actin, α_1A_-, α_1B_-, and α_1D_-AR PCR products were 353, 502, 530, and 540 bp, respectively.

PCR was carried out with Platinum *Taq *DNA polymerase (Invitrogen) in a PCR Express (Hybaid Ltd., United Kingdom) thermal cycler. The amplification reactions, repeated for 35 cycles, consisted of denaturation at 94°C for 1 minute, annealing at 55°C for 30 seconds, and extension at 72°C for 1 minute. PCR products were run on a 1.4% agarose gel.

### Determination of intracellular calcium

Human aortic smooth muscle cells were loaded for 1 hour with 5:M Calcium Green ™ 1 AM (Molecular Probes, Eugene, OR). In certain experiments, Rat 1 fibroblasts were also loaded with the Calcium Green. Cells were then washed twice with serum containing medium and visualized with an inverted microscope with a Xe arc lamp with a Plan-Apo 60X oil immersion objective and an excitation filter of 480/15 nm and an emission filter of 535/20 nm. Images were taken using a CoolSnap HQ camera. Indicator dye-loaded cells underwent several drug treatments. Human aortic smooth muscle cells were pre-treated with vehicle, 1 nM prazosin or 30 nM BMY 7378 for 20 minutes, followed by 25 :M phenylephrine. Stably transfected fibroblasts were challenged with 10 :M phenylephrine. A higher phenylephrine concentration was required in smooth muscle to observe an equivalent increase in the calcium signal. Images from calcium measurements were processed using Metamorph software (Molecular Devices, Sunnyvale, CA). In the analysis, the nucleus was masked leaving the cytoplasm. The mean intensity of the cytoplasm was then taken for each cell. Images were prepared using Adobe Photoshop version 6.0 (Adobe Systems, Mountain View, CA). lmaging data were analyzed by one-way analysis of variance with Tukey's post-hoc test using GraphPad Prism version 3.00 for Windows (GraphPad Software, San Diego, CA). In all figures, the data are expressed as the mean and standard error of the mean (S.E.). A value of P < 0.05 was considered statistically significant.

### Reactive oxygen species

The generation of reactive oxygen species was measured using Mitotracker ROS (Invitrogen, Carlsbad, CA) diluted in DMSO. Human aortic smooth muscle cells, attached to glass coverslips, were incubated for 20 min at 37° with 5 nM Mitotracker ROS diluted in Serum Free Medium. Cells were washed twice with medium and fresh medium applied. After this time 10 μM phenylephrine was added and incubated with the cells for 20 min. Preliminary studies established this time as that optimal to record a significant increase in ROS levels. In certain experiments, cells were pretreated with 1 nM prazosin or 30 nM BMY 7378 prior to the addition of phenylephrine. After agonist incubation, the cells were fixed with 1.0% formaldehyde in PBS for 10 min and washed with PBS. Cells were mounted on slides with Vectashield (Vector Laboratories, Burlingame, CA). Cells were imaged by confocal microscopy as described above. Images were processed using Metamorph software (Molecular Devices, Sunnyvale, CA). Images were prepared using Adobe Photoshop version 6.0 (Adobe Systems, Mountain View, CA). lmaging data were analyzed by one-way analysis of variance with Tukey's post-hoc test using GraphPad Prism version 3.00 for Windows (GraphPad Software, San Diego, CA). In all figures, the data are expressed as the mean and standard error of the mean (S.E.). A value of P < 0.05 was considered statistically significant.

### Assessment of aortic contractile function

All animal protocols were reviewed and approved by the University of Kentucky Institutional Animal Care and Use Committee. Isolated blood vessels were prepared by techniques routinely used in our laboratory [21,22,27,28]. Aorta were removed from male Sprague-Dawley rats, cleaned of adventitia and extraneous tissue and then segmented into 1–2 mm rings. Following isolation, aortic rings were placed in a 37EC cell culture incubator and treated for 12 hr with 1 :M albuterol, a selective β_2_-AR agonist or a vehicle control. After this period, rings were placed in the tissue baths for the assessment of contractile activity. The water-jacketed muscle baths were filled with physiologic saline solution maintained at 37°C with constant oxygenation (95% O_2_, 5% CO_2_, pH 7.4) and under a passive force of 2.0 grams. Previous studies have shown that this passive force gives optimal responses. Aortic rings were contracted with 25 mM KCl and the ability of increasing amounts of albuterol to induce aortic relaxation was measured. Phenylephrine-dose response curves were also generated in a separate set of aortic rings treated with albuterol. Changes in the force generation were recorded using force displacement transducers (Astro-Med, Inc., Grass Instruments, West Warwick, RI) interfaced to a Dell computer. Data were retrieved using PolyVIEW version 2.5 and analyzed using GraphPad Prism version 3.00 for Windows (GraphPad Software, San Diego, CA).

## Abbreviations

AR: Adrenergic receptor; GPCR: G-Protein-Coupled Receptor; HASMC: Human aortic smooth muscle cell; GFP: Green fluorescent protein.

## Authors' contributions

MLG performed or assisted in many of the studies in the manuscript. Specifically, figures 1, 3, 4 and 6. Also, wrote the manuscript. JLS mantained cell culture, performed or assisted in studies reported in figures 1, 3, 4, 5. Edited the manuscript and performed statistical analysis. KAO performed the PCR analysis in figure 2. DFM supervised the PCR studies in figure 2. LAS assisted in calcium studies in figures 3 and 4. RWH supervised calcium imaging studies in figures 3 and 4. SDK supervised preparation and purification of adenoviral vectors. MTP supervised MLG and JLS and is senior author. Edited manuscript and oversaw all aspects of this work. All Authors read and approve of this work.
